# Socioeconomic differences in body mass index in Spain: An intersectional multilevel analysis of individual heterogeneity and discriminatory accuracy

**DOI:** 10.1371/journal.pone.0208624

**Published:** 2018-12-10

**Authors:** Aránzazu Hernández-Yumar, Maria Wemrell, Ignacio Abásolo Alessón, Beatriz González López-Valcárcel, George Leckie, Juan Merlo

**Affiliations:** 1 Departamento de Economía Aplicada y Métodos Cuantitativos, Facultad de Economía, Empresa y Turismo, Universidad de La Laguna (ULL), San Cristóbal de La Laguna, Santa Cruz de Tenerife, España; 2 Unit for Social Epidemiology, Faculty of Medicine, Lund University, Malmö, Sweden; 3 Department of Gender Studies, Lund University, Lund, Sweden; 4 Departamento de Métodos Cuantitativos en Economía y Gestión, Universidad de Las Palmas de Gran Canaria (ULPGC), Las Palmas de Gran Canaria, España; 5 Centre for Multilevel Modelling, University of Bristol, Bristol, United Kingdom; 6 Centre for Primary Health Care Research, Region Skåne, Malmö, Sweden; McMaster University, CANADA

## Abstract

Many studies have demonstrated the existence of simple, unidimensional socioeconomic gradients in body mass index (BMI). However, in the present paper we move beyond such traditional analyses by simultaneously considering multiple demographic and socioeconomic dimensions. Using the Spanish National Health Survey 2011–2012, we apply intersectionality theory and multilevel analysis of individual heterogeneity and discriminatory accuracy (MAIHDA) to analyze 14,190 adults nested within 108 intersectional strata defined by combining categories of gender, age, income, educational achievement and living situation. We develop two multilevel models to obtain information on stratum-specific BMI averages and the degree of clustering of BMI within strata expressed by the intra-class correlation coefficient (ICC). The first model is a simple variance components analysis that provides a detailed mapping of the BMI disparities in the population and measures the accuracy of stratum membership to predict individual BMI. The second model includes the variables used to define the intersectional strata as a way to identify stratum-specific interactions. The first model suggests moderate but meaningful clustering of individual BMI within the intersectional strata (ICC = 12.4%). Compared with the population average (BMI = 26.07 Kg/m2), the stratum of cohabiting 18-35-year-old females with medium income and high education presents the lowest BMI (-3.7 Kg/m2), while cohabiting 36-64-year-old females with low income and low education show the highest BMI (+2.6 Kg/m2). In the second model, the ICC falls to 1.9%, suggesting the existence of only very small stratum specific interaction effects. We confirm the existence of a socioeconomic gradient in BMI. Compared with traditional analyses, the intersectional MAIHDA approach provides a better mapping of socioeconomic and demographic inequalities in BMI. Because of the moderate clustering, public health policies aiming to reduce BMI in Spain should not solely focus on the intersectional strata with the highest BMI, but should also consider whole population polices.

## Introduction

### Socioeconomic differences in body mass index (BMI) in Spain

Obesity is a non-communicable disease considered to be the grand pandemic of the 21^st^ century [1], and is especially prevalent in developed countries [2], while also increasing in middle- and low-income countries [3]. As a result, obesity is one of the most important public health challenges for Europe [4, 5]. According to the World Health Organization (WHO), in 2016 more than 1.9 billion adults worldwide (or 39% of the total adult population) were overweight, and over 650 million of these (or 13% of the population) were obese [3]. As in many countries belonging to the Organization for Economic Co-operation and Development (OECD) [6], this health problem is rapidly worsening in Spain [7, 8]. For instance, according to the Spanish National Health Survey (SNHS), the prevalence of adult obesity rose from about 7% in 1987 to approximately 15% in 2006 [9] and 17% in 2012 [10], while according to the Study on Nutrition and Cardiovascular Risk in Spain (ENRICA), which used direct anthropometric measurements [11], 23% of the Spanish adult population was categorized as obese in 2008–2010 [12].

Additionally, many studies have pointed to the existence of a socioeconomic gradient of obesity in Spain [8, 9, 13–16], as well as in many other countries [17–20]. There is a general consensus that obesity is associated with lower levels of education and income [8, 12, 21–24]. In addition, the prevalence of obesity appears to have increased more rapidly among less privileged groups [25, 26]. However, most traditional studies have investigated socioeconomic gradients defined by separate or mutually adjusted individual variables, such as income or education [12, 14, 18–20, 22, 23, 27, 28]. These studies are currently being criticized [29–31] as they provide a rather simplistic mapping of health inequalities and, therefore, they fail to capture appropriately the heterogeneous influence of socioeconomics factors on individual health outcomes. In addition, such studies tend to conceptualize measures of socioeconomic position, like education and income, as individual, private characteristics, rather than indicators of a socioeconomic context that influences individual BMI over and above individual-level characteristics. Furthermore, traditional studies do not provide information about the possible existence of interaction effects between different socioeconomic variables, and they are based on differences between group averages, ignoring the typically substantial variation in BMI around these averages.

Recently, however, an innovative conceptual and methodological approach has been introduced [32] and developed to investigate socioeconomic and demographic differences in BMI in the USA [33], and chronic obstructive pulmonary disease (COPD) incidence in Sweden [34]. This approach is based on the integration of intersectionality theory and multilevel analysis of individual heterogeneity and discriminatory accuracy (MAIHDA) and provides a superior framework for the study of disparities in health [29, 30].

### Intersectionality theory: Categorical and anti-categorical perspectives

Intersectionality theory, which largely builds on the seminal work of Crenshaw and other scholars [35–41], implores the conceptualization of categories such as race/ethnicity, sex/gender, class and sexual orientation not as separate but as interlocking. Power structures are set at the center of the analysis, as focus is directed to what social categories and their interactions can disclose about systems of privilege and disadvantage [38]. Intersectionality thereby offers a theoretical framework that can direct epidemiological attention beyond unidimensional social gradients toward more complex interactions between axes of social differentiation, and toward structural motors for inequalities, rather than individual-level behaviors and risk factors [42]. In recent years, intersectionality has been advocated and, to a certain extent, integrated [29–31, 37, 43] in epidemiological studies for the purpose of developing the understanding of health inequities. Although the intersectional approach also has a presence in economic research [44–47], as far we know, the application of this kind of study is not yet common in the health economics field [48, 49].

As described by McCall [41], three viewpoints exist within intersectionality research: the *anticategorical*, the *intercategorical (or categorical)* and the *intracategorical*. The anticategorical approach is grounded in the insight that categorizations do not capture the complexities of social life, and that the use of social categorizations may only perpetuate the existing power structures of which such categorizations form part. An anticategorical approach therefore consists of questioning or deconstructing categorizations, as a way of working toward deconstructing inequality itself. On the other hand, the intercategorical approach involves the provisional adoption of social categories in order to document inequalities among strata. It addresses the fact that inequality exists within society, and uses intersectionality to analyze this inequality [41]. Finally, the intracategorical approach tends to “focus on particular social groups at neglected points of intersection…in order to reveal the complexity of lived experiences within such groups” [41] (p.1774). The intracategorical approach has largely been operationalized within a qualitative framework, but is less applicable in quantitative health inequalities research based on comparison between groups. Therefore, in the rest of our paper we just distinguish between categorical and anticategorical perspectives.

Most intersectional research so far builds on qualitative methodology. However, the idea of categorical intersectionality fits very well with quantitative, epidemiological measurement. In fact, the vast majority of intersectional approaches applied in quantitative analyses are generally of this categorical type [50–52], and typically consist of measurement of between-group differences in average risk. There is, however, a fundamental difference between qualitative analysis in social sciences and positivistic quantitative epidemiology when it comes to the application of the intersectional framework [29]. Qualitative intersectionality research typically aims to provide in-depth knowledge about intersecting social relationships and power structures, while attending to experienced, interpretive, historical or subjective aspects of social categorizations or identities, which are not easily captured in statistical models [39]. Moreover, the perhaps most basic tenet of intersectionality is the emphasis on the fluidity and complex interrelatedness of social categorizations, which stands at odds with the neat decomposition of separable additive effects of variables that is typically a feature of quantitative analysis [53]. Intersectionality theory has understood—and so denominated—the effect differences between intersectional categories as interactions. However, being a social theoretical qualitative approach, it does not formally distinguished between the notion of additive and interactive effects as applied in quantitative epidemiological research. Nevertheless, as discussed elsewhere [29], from the perspective of social epidemiology, the positivistic quantitative decomposition and analysis of intersectional groups, as well as the analysis and validation of intercategorical (or just categorical) and anticategorical approaches to intersectionality, seems very possible. In epidemiology, the term interaction (of effects) has a very specific meaning. On the additive scale, interaction refers to a departure from additivity. That is, the observed effect is larger than the sum of the additive component effects. Our approach refers to intersectional effect as those observed at the strata level, while we are, however, aware that these can be decomposed into “additive” and “interactive” intersectional effects. Drawing on previous research stressing the capacity of categorizations to discriminate with accuracy between those that present or not a certain health outcome [54–60], we have proposed a methodology for evaluating both anti-categorical and categorical approaches [29, 30, 61]. Previous intersectional studies have applied measures of discriminatory accuracy such as the area under the receiver operating characteristic (ROC) curve of intersectional strata entered into conventional fixed effects logistic regression models [61]. Analogously, the use of components of variance in intersectional MAIHDA analysis provides valuable information that may help to discern whether or not the intersectional strata represent meaningful contexts that condition the individual health outcome [29].

### Intersectional MAIHDA

Multilevel modeling has been identified as a promising way forward for intersectionality in epidemiology [29, 30, 33]. Many readers will recognize multilevel regression analysis from the research domain of neighborhoods and health, where individuals are nested within contexts defined by geographical administrative boundaries. The innovative aspect of applying MAIHDA on intersectional strata is that the individual health outcome (i.e., BMI) is modeled using a multilevel regression analysis of individuals nested within the cells of a matrix defined by all possible combinations of socioeconomic and demographic variables [29].

Analyzing an intersectional matrix by means of MAIHDA confers multiple advantages for the investigation of socioeconomic differences in health [29, 33], including: *(i*) the intersectional matrix itself provides a detailed mapping of the socioeconomic differences in average BMI and (*ii*) the multilevel analysis of the intersectional matrix provides precision-weighted estimates of the average BMI for each intersectional group (i.e., empirical Bayes, posterior, or shrunken predictions), which allows for the inclusion of small size strata. The MAIHDA informs researchers about (*iii*) the existence of stratum specific interaction of effects and (*iv*) it also decomposes the individual heterogeneity in BMI into within and between intersectional group components. This variance component analysis allows researchers to investigate the discriminatory accuracy of the intersectional strata and to quantify the size of the “general contextual effects” [29]. That is, “the influence of the context itself without specifying any other contextual characteristics than the very boundaries defining it” (see elsewhere [29], section 2). So, our study avoids what has been referred to as the “tyranny of the averages” in epidemiology [54–56], meaning attribution of the average outcome to all individuals in a group without considering the individual heterogeneity of outcomes around that average. Most relevant, (*v*) the MAIHDA considers the dimensions of social identity and position as contexts rather than as individual characteristics. In this way, the risk of “blaming the victim” decreases, as the interpretational focus of BMI differences between strata is directed to societal factors. In addition, the intersectional MAIHDA approach provides (*vi*) improved scalability for higher dimensions (i.e., a higher capability to accommodate many intersectional strata), and (*vii*) better model parsimony (i.e., intersectional strata are modeled with only one random effects parameter rather than as a separate coefficient for every stratum).

On top of these seven arguments, another major reason for the use of MAIHDA is that the multilevel analytical approach does not dislocate the individual from the population (i.e., intersectional category) variance [29]. Rather, the analyses consider the total individual variance as a continuum that can be decomposed at different levels of the analysis, thus enabling the simultaneous exploration of both between-group and within-group components of individual heterogeneity. Group effects are, thereby, appraised not only through the assessment of differences between stratum averages, but also through the gauging of the share of the individual heterogeneity (i.e., variance in BMI) that exists at the group level [30, 62–64]. This is the basis of the concept of intraclass correlation coefficient (ICC) or, in more general terms, of the variance partition coefficient (VPC) that is used for the assessment of clustering or of general contextual effects in multilevel regression models [62, 65–67]. The VPC measures the share of the total individual variance that is located at the group or category level and, therefore, it is also a measure of discriminatory accuracy that discerns the accuracy of the categories for classifying individuals [68].

In traditional studies, the theory and a priori hypotheses justifying the investigation of the association between simple measures of socioeconomic position (e.g., education or income) and health-related outcomes are well established [69]. Therefore, most traditional studies of health inequalities are deductive even if the hypotheses are not explicitly defined in the study. However, in the analysis of an intersectional matrix we do not necessarily have an established hypothesis for each of the intersectional groups. Nevertheless, in spite of this explorative nature, the intersectional MAIHDA approach provides worthy inductive information on socioeconomic differences in health, reaching beyond traditional studies of health inequalities. This new methodology increases our understanding of the dynamics of privilege and disadvantage that drive the production of health disparities, which is not only interesting from an epidemiological point of view, but also from an economic one.

### Aims

In summary, the principal aim of our study is to investigate socioeconomic differences in BMI in Spain. In order to do so, we move beyond the study of unidimensional social gradients in BMI toward the application of MAIHDA [30, 63]. Our hypothesis is that our understanding of the socioeconomic heterogeneity of BMI can be improved through the consideration not only of socioeconomic gradients defined by separate or mutually adjusted individual variables, such as income or education, but also by considering the combination of different axes of social differentiation. This intersectional approach better captures the interlocking systems of privilege and disadvantage in society than does the traditional analysis of socioeconomic disparities, which we are habituated to. In this way, we aim to provide an improved mapping and documentation of the distribution of social, economic and demographic disparities of BMI in Spain.

## Population and methods

### Study population

This study is based on microdata from the Spanish National Health Survey (SNHS) performed during 2011–2012 [70]. The SNHS is a cross-sectional survey based on personal interviews with 21,007 individuals aged 15 or higher, residing in Spain at the time of the survey. The survey was carried out by the Spanish National Institute of Statistics in collaboration with the Ministry of Health, Social Services and Equality. The respondents were selected via a stratified three-stage sample, through which municipalities, households and members of households were randomly selected. See elsewhere for detailed information on the survey [70].

**Fig 1:**
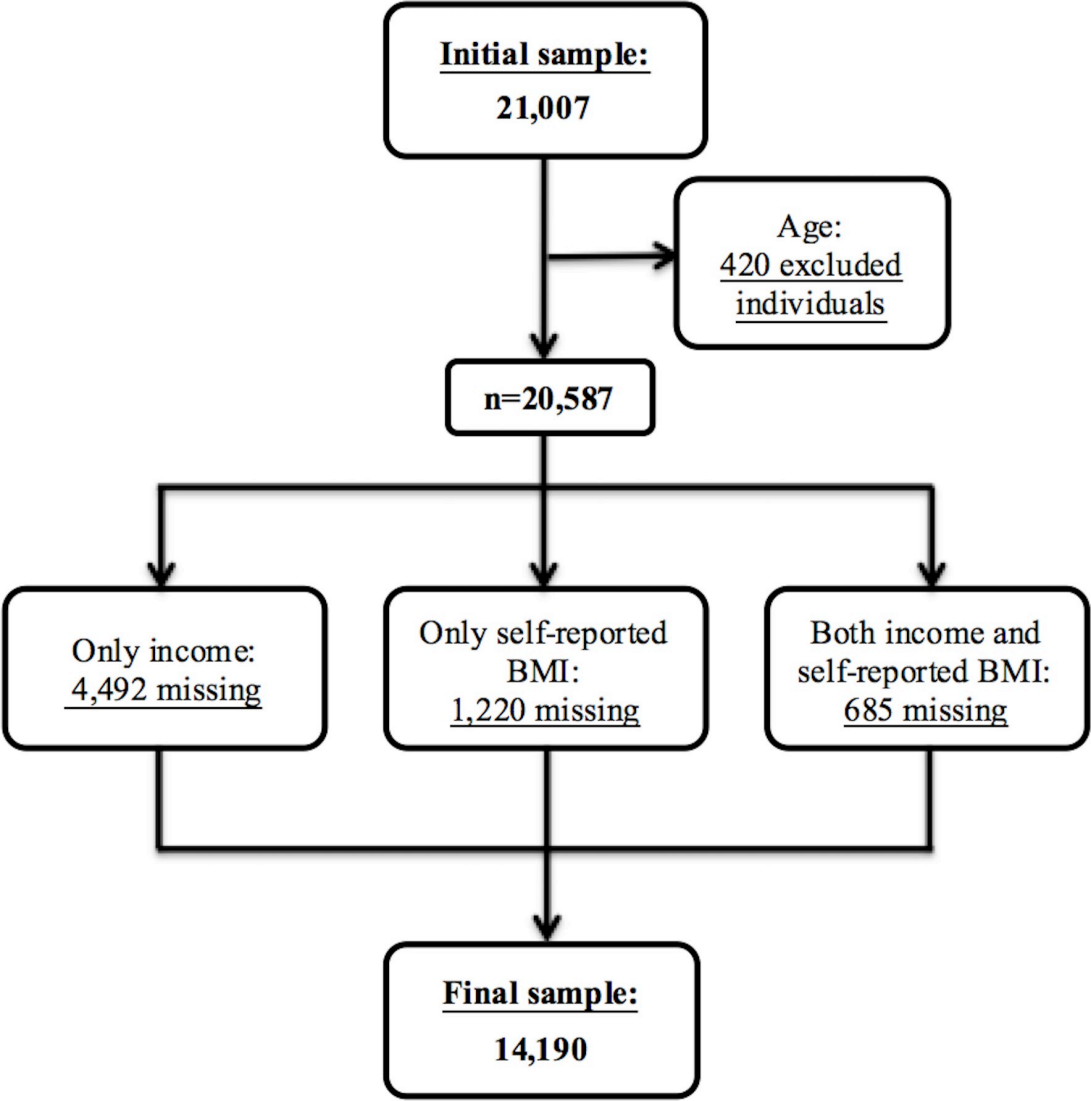
Flowchart indicating the exclusion criteria and the number of individuals excluded from the study sample.

From the 21,007 individuals initially included in the database, we excluded 420 individuals aged 15–17 years, as our study focuses on the adult population (*≥*18 years). In addition, 6,397 individuals were excluded due to missing information on only income (70%), only self-reported BMI (19%) and both income and self-reported BMI (11%) (Fig 1). The remaining variables–gender, education, and living alone–do not contain any missing values. The final sample consists of 14,190 interviewees, corresponding to 68% of the original adult sample. We return to discuss potential implications of both missingness and the self-reported nature of BMI for our findings in the Discussion.

### Assessment of variables

Several social dimensions influence health status and generate health inequalities [71]. Some of them may particularly affect the BMI of the population in Spain [8, 9, 13, 15, 16, 72]. We have selected the following demographic and socioeconomic determinants to evaluate their effects on the BMI distribution among the Spanish population.

#### Body mass index

The continuous dependent variable is BMI, calculated from self-reported measures of weight and height, as weight in kilograms divided by the square of height in meters (kg/m^2^).

#### Gender

We categorized gender as *male* or *female*, as these were the only two alternatives included in the survey. We defined male as the reference category.

#### Age

We categorized age into three groups: *18–35 years*, *36–64 years* and *≥65 years* (up to 103). This age categorization is based on the assumption that individuals in each age group to some degree share similar health and life conditions [73]. In addition, these categories allow for classification of individuals into young, middle-aged and older adults [73]. The youngest age group was set as the reference category.

#### Income

Income refers to the net monthly income of the household. It is provided in the survey. Income is measured in euros and presented in ten bands defined by the SNHS: (i) ≤550 euros; (ii) 551–800; (iii) 801–1,050; (iv) 1,051–1,300; (v) 1,301–1,550; (vi) 1,551–1,850; (vii) 1,851–2,250; (viii) 2,251–2,700; (ix) 2,701–3,450; and (x) >3,450 euros [74]. We account for the differing number of individuals per household and the economies of scale which likely arise in larger households as follows. First, we define a continuous measure of household income by assigning the midpoint of the relevant income band for each household as its income. While the last interval is open, we assumed that its range is the same as that in the preceding interval, as done elsewhere [8]. Then, we calculated the equivalent household income by dividing the household income by the weighted number of individuals in the household, applying the OECD weights of 1 for the reference adult, 0.5 for each additional adult, and 0.3 for each child [75]. Thereafter, we divided individuals into three income groups by tertiles: *low* (lowest to 1^st^ tertile), *medium* (> 1^st^ tertile to 2^nd^ tertile) and *high* (>2^nd^ tertile). We used the highest level of income as the reference category in the comparisons.

#### Education

We assumed that the socioeconomic position of the individual is given by the household where the individual resides. Therefore, the educational achievement variable refers to the highest educational level present in the household rather than to that of the individual themselves. The survey questionnaire classifies educational achievement into nine levels, ranging from illiteracy to completed university studies. The categorization was made as follows: (i) not applicable, s/he is under 10 years old; (ii) cannot read or write; (iii) incomplete primary education (attended school for less than five years); (iv) primary education (attended school for five years or more, but did not reach the last year of compulsory education); (v) first stage of secondary education (elementary secondary education or similar); (vi) upper secondary education; (vii) intermediate-level professional education or equivalent; (viii) advanced professional training or equivalent; and (ix) university studies or equivalent [70]. Based on the Classification of Programmes at Educational Levels (CNED-P 2014) [76], within the National Classification of Education, we collapsed these nine levels into three bands of educational achievement: *low* (ii—iv), *medium* (v—viii) and *high* (ix). However, as a distinction is merited between advanced professional training or equivalent (viii) and university studies or equivalent (ix), we have grouped these separately. We use the high level of educational achievement as the reference category in the comparisons.

#### Living alone

As discussed elsewhere [72], civil status is a BMI determinant, and we assume that the condition of living alone has a considerable influence on the BMI. Therefore, using detailed information on household composition from the household questionnaire [74], we classified individuals into *living alone* (i.e., single-person household) or *cohabiting* (i.e., multi-person household). We used the cohabiting category as the reference category in the comparisons.

#### Intersectional strata

In order to consider simultaneously multiple axes of social differentiation, and the potential interaction between these, we created 2×3×3×3×2 = 108 intersectional strata corresponding to different theoretical combinations of gender (2 categories), age (3 categories), income (3 categories), education (3 categories), and living alone (2 categories). Two of these strata were empty and 31 were rather small (i.e., less than 30 individuals) (see S1 Table). As proposed by Evans et al [33], we denominated these categories as “intersectional strata” rather than intersectional categories or intersectional groups. By doing so, we aim to avoid the reification of categorical labels and the danger of treating social labels as unchanging and inflexible.

### Statistical analysis

We carried out an intersectional MAIHDA analysis with individuals at the first level of analysis, and the intersectional strata at the second level [29, 32, 33]. We performed two consecutive intersectional multilevel linear regression analyses.

#### The first or “simple intersectional” model

The first multilevel model only includes a random intercept for the intersectional strata and partitions the total variance in BMI between the two levels, i.e., between and within intersectional strata. This model is therefore a “null”, “empty” or “variance components” model. The model can be written as

$$y_{ij}=\beta_{0}+u_{j}+e_{ij}$$ (1)

where *y*_*ij*_ denotes the BMI of individual *i* (*i* = 1,…,*n*_*j*_) in intersectional stratum *j* (*j* = 1,…,*J*), *β*_0_ denotes the intercept, *u*_*j*_ denotes the stratum-level random effect, and *e*_*ij*_ denotes the individual-level residual. The intercept measures the overall population average BMI across all strata and individuals. The stratum random effects measure the difference between the average BMI in each stratum and the overall population average BMI. The individual residuals measure the difference between the BMI of each individual and the average for their stratum.

The stratum random effects are assumed to follow a normal distribution with mean 0 and variance $\sigma_{u}^{2}$ around the overall population average BMI.

$$u_{j}∼N(0,\sigma_{u}^{2})$$ (2)

The individual residuals are assumed to follow a normal distribution with mean 0 and variance $\sigma_{e}^{2}$.

$$e_{ij}∼N(0,\sigma_{e}^{2})$$ (3)

The *u*_*j*_ are not model parameters. Rather, values are assigned to the *u*_*j*_ post-estimation. These values are so-called empirical Bayes, posterior, or shrinkage predictions of the differences between the average BMI in each stratum and the overall population average BMI. Essentially, the observed differences are shrunk towards zero as decreasing function of stratum size in recognition that observed differences calculated for small strata will in general be less precise than observed differences calculated for larger strata due to the smaller number of individuals on which they are based. Put differently, all else equal, smaller strata will disproportionally display the most extreme observed differences. Shrinkage pulls the observed differences of smaller strata towards the overall average as they are based on so little data and so shrinkage therefore protects us from over interpreting their disproportionately extreme values (see [77] for more information).

The simple intersectional model decomposes the total individual variance into its variance components: $\sigma_{u}^{2}$ and $\sigma_{e}^{2}$. This allows the calculation of the variance partition coefficient defined as the share of the total individual variance that is between the intersectional strata level. In other words, the VPC quantifies the importance of intersectional effects in predicting BMI.

$$VPC\equiv ICC=\frac{\sigma_{u}^{2}}{\sigma_{u}^{2}+\sigma_{e}^{2}}$$ (4)

This VPC can also be interpreted as an intra-class correlation coefficient. This ICC measures the correlation in BMI between two individuals randomly selected from the same intersectional stratum. The larger the VPC and ICC, the larger the share of individual differences in BMI attributable to intersectional strata, or in other words, the stronger the clustering in BMI within the strata and the larger the general contextual effect of the intersectional strata (see elsewhere [29], section 2). If the VPC and ICC are low, the intersectional strata are not very relevant for understanding individual differences in BMI, while the opposite is true if these statistics are high. Therefore, the VPC and ICC assess the validity of intersectional strata (i.e., “categories”) as social constructs for understanding individual inequalities in BMI [29, 30]. Within an intersectionality framework, then, low VPC and ICC merit an anti-categorical [41] perspective while high VPC and ICC corresponds with a categorical [41] interpretation.

#### The second or “intersectional interaction” model

The second model provides an innovative approach to investigating the existence of any two- or higher-way interaction effects between the variables used to define the intersectional strata. The intersectional framework directs attention towards interaction or interactive effects, as the effect of each intersectional stratum may be larger (or smaller) than the sum of the main effects (additive effects) of the different dimensions defining the intersectional stratum. For instance, the effect on BMI of being a young, highly educated, rich woman living alone might be different to the sum of the main effects of each variable. In our first simple intersectional model, the effects of the intersectional categories on BMI is expressed by the stratum random effects *u*_*j*_, but these effects conflate the main effects of the variables defining the strata and any interaction effects between these variables (see elsewhere [29], section 6.2). In order to isolate the interaction effects, our second intersectional interaction model adjusts for the very variables used to construct the intersectional strata. This is done through entering each variable into the model as a series of dummy variables, one for each category, where in each case we omit the reference category, as previously defined. The model can therefore be written as

$$y_{ij}=\beta_{0}+\beta_{1}x_{1j}+\beta_{2}x_{2j}+\beta_{3}x_{3j}+\beta_{4}x_{4j}+\beta_{5}x_{5j}+\beta_{6}x_{6j}+\beta_{7}x_{7j}+\beta_{8}x_{8j}+u_{j}+e_{ij}$$ (5)

where *x*_1*j*_ is a dummy variable for female, *x*_2*j*_ and *x*_3*j*_ are dummy variables for the middle and old age groups, *x*_4*j*_ and *x*_5*j*_ are dummy variables for the low and middle income groups, *x*_6*j*_ and *x*_7*j*_ are dummy variables for the low and middle education groups, and *x*_8*j*_ is a dummy variable for living alone. The intercept, *β*_0_ measures the predicted BMI of the stratum defined when all the dummy variables equal zero (i.e., the reference individuals: 18 to 35-year-old males, with high income and high education and who cohabit). The regression coefficients measure the mean effect of each individual characteristic.

While the *u*_*j*_ in our first simple intersectional model conveys the total or “ceiling” effect of the intersectional strata (intersectional effects) due to both main effects (additive effects) and interactions (interactive effects), the *u*_*j*_ values in this second intersectional interaction model are only attributable to the interaction effects existing between the combinations of the variables that define the strata, once the model have been adjusted for the main effects of these social and demographic dimensions.

It should be observed that in the intersectional interaction model, the subscripts of the variables that define the strata are “*j*” and not “ *ij*” as would usually be expected for individual level variables in traditional multilevel analyses of individual outcomes. This is because these characteristics are actually considered to be contextual and are, therefore, constant for each intersectional combination. That is, the categories of the variables do not vary within the intersectional strata.

If the *u*_*j*_ in our first simple intersectional model were only due to the additive effects of the variables that construct the intersectional strata, adjustment for these variables in this second model would completely explain the variance between the intersectional strata. Therefore, $\sigma_{u}^{2}$, all the *u*_*j*_, and the conditional ICC would each be estimated to be 0. However, in the presence of any interaction effects, we would observe some residual between stratum variation and $\sigma_{u}^{2}$, the *u*_*j*_, and the conditional ICC would each be estimated to be greater than 0. The stratum random effects *u*_*j*_ in the intersectional interaction model capture the difference between the average BMI of each specific intersectional stratum and the predicted BMI for each stratum based only on the main effects of the variables. They express, in other words, the effects beyond what is expected based on the contributions of the additive effects of the variables that define the intersectional category.

As discussed by Evans et al [33], it should be noted that the choice of a specific reference category for the variables included as fixed effects covariates in the intersectional interaction model does not influence the value of the *u*_*j*_’s. Classical interaction studies which model the interaction between variables by entering their products as covariates normally use specific categories as references. However, the intersectional interaction model highlights the existence of an interaction in relation to the combination of variables that compose the stratum, while allowing us to determine simultaneously whether or not intersectional strata in general exhibit evidence of intersectional interaction [33].

#### Model estimation

The models were estimated using Markov chain Monte Carlo (MCMC) methods as implemented in the MLwiN multilevel modeling software [78, 79]. We called MLwiN from within Stata using the user-written runmlwin command [80]. We specified diffuse (vague, flat, or minimally informative) prior distributions for all parameters. For each model, we specified a burn-in length of 5,000 iterations and a monitoring chain length of 10,000 iterations. Visual assessments of the parameter chains and standard MCMC convergence diagnostics suggested that the lengths of these periods were adequate. We calculated the Bayesian deviance information criterion (DIC) as a measure of the goodness of fit of our models [78]. Models with smaller DIC are preferred to models with larger DIC, with differences of five or more considered to be substantial [81].

## Results

Table 1 indicates that the average self-reported BMI in Spain, in 2011–12, was 26.24 kg/m^2^. We can also observe that there were gender differences, as men presented on average a 1.25 units higher BMI than women. In addition, the average BMI increased with age and was higher among people with low income and low educational achievement, compared to people with high income and high educational achievement respectively.

**Table 1:** Mean Body Mass Index (BMI) and (95% confidence interval) in Kg/m2 reported separately by gender, age, income, education, and living alone.

		Number of individuals (%)	Mean BMI (95% CI)
	Overall	14,190 (100)	26.24 (26.17–26.32)
Gender	Males	6,821 (48.07)	26.89 (26.79–26.98)
	Females	7,369 (51.93)	25.64 (25.53–25.76)
Age	≤35	3,078 (21.69)	24.37 (24.22–24.52)
	36–64	7,386 (52.05)	26.41 (26.31–26.51)
	≥65	3,726 (26.26)	27.45 (27.32–27.59)
Income	Low	5,619 (39.60)	26.90 (26.78–27.03)
	Medium	3,886 (27.39)	26.20 (26.06–26.34)
	High	4,685 (33.02)	25.48 (25.36–25.60)
Education	Low	2,521 (17.77)	27.76 (27.58–27.93)
	Medium	8,268 (58.27)	26.28 (26.18–26.38)
	High	3,401 (23.97)	25.03 (24.89–25.16)
Living alone	No	10,971 (77.32)	26.20 (26.11–26.28)
	Yes	3,219 (22.68)	26.39 (26.23–26.55)

The results from the intersectional MAIHDA are showed in Table 2. The first simple intersectional model shows the existence of a clustering of individual BMI within the intersectional strata: 12.4% of the total individual variance in BMI is located at the intersectional strata level.

**Table 2:** Results from the multilevel linear regression analysis.

		Model 1 Simple intersectional	Model 2 Intersectional interaction
**Measures of association**			
Intercept		26.07 (25.76–26.38)	25.18 (24.55–25.82)
Gender	Males		Ref.
	Females		-1.16 (-1.49 –-0.85)
Age (years)	≤35		Ref.
	36–64		2.12 (1.72–2.53)
	≥65		2.61 (2.21–3.04)
Income	Low		0.71 (0.34–1.09)
	Medium		0.20 (-0.18–0.59)
	High		Ref.
Education	Low		1.71 (1.26–2.15)
	Medium		0.96 (0.59–1.37)
	High		Ref.
Living alone	No		Ref.
	Yes		-0.50 (-0.84 –-0.19)
**Measures of variance**			
Variance level 2:	Intersectional strata	2.55 (1.83–3.52)	0.35 (0.21–0.53)
Variance level 1:	Individuals	18.10 (17.67–18.53)	18.09 (17.67–18.51)
ICC		12.4%	1.9%
DIC		81,451.49	81,413.96

**Fig 2:**
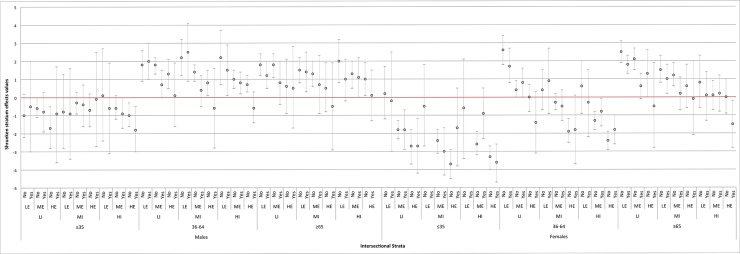
Differences between the estimated average BMI in each intersectional stratum and the overall population average BMI (i.e., shrunken stratum effects values from the simple intersectional model 1). The intersectional strata are ordered according to the demographic and socioeconomic dimensions used to define the strata. The red horizontal line at value 0 corresponds with the population average BMI. The exact values and the specific definition of the intersectional strata are indicated in the S1 Table. LI: Low Income; MI: Medium Income; HI: High Income. LE: Low Education; ME: Medium Education; HE: High Education.

Fig 2 shows the predicted differences between the average BMI of the intersectional strata and the population average BMI (i.e., the shrunken stratum effects) obtained from the simple intersectional model (model 1). Compared with the population average BMI, 19 intersectional strata have a significantly lower average BMI and 30 a significantly higher average BMI (their 95% confidence intervals do not overlap with the population average BMI) (Fig 2 and S1 Table). We can notice that the intersectional stratum of *females who are 18 to 35 years of age*, *have medium income*, *high education and do not live alone* presented the largest negative BMI difference from the average (i.e., -3.7 BMI units). At the opposite end, the stratum made up of *females between 36 and 64 years*, *who have low income*, *low education and do not live alone* showed an average BMI that is 2.6 units higher than the population average (see the S1 Table).

The simple intersectional model and the predicted stratum effects, however, do not distinguish the additive effects of the five variables defining the strata from any interaction effects. This information can be disentangled in the intersectional interaction model (model 2). The fixed-effects regression coefficients report the main or additive effects of these variables. The results show women had an average BMI 1.16 units lower than that of men, having controlled for the other variables. Moreover, people who lived alone had average BMI that was 0.50 units lower than those who cohabited, all else being equal. It is also shown that being over 35 years of age results in a notable increment in average BMI, particularly from the age of 65 (i.e., 2.61 BMI units more than the reference category, individuals ≤35 years). In addition, average BMI increases as income and, particularly, education declines. People with low income have an average BMI 0.71 units higher than those with high income, all else equal, while individuals with low educational attainment present a mean BMI 1.71 units higher than those with higher educational attainment.

As expected, the inclusion of main effects in model 2 (Table 2) reduced the stratum variance considerably, from 2.55 to 0.35. Thus, 86% of the variation across strata is attributable to the main effects of the five variables (= 100×(2.55−0.35)/2.55), while 14% is attributable to two- and higher-way interaction effects between these variables. The reduction in the stratum variance leads to a reduction of the ICC from 12.4% to 1.9%. This small conditional ICC means that 1.9% of the remaining variation in individual BMI is due to interaction effects between the five variables used to define the intersectional strata. Thus, interaction effects appear to play a small role in explaining why individuals vary in their BMI; the vast majority of variation is attributable to other variables not recorded in our study.

**Fig 3:**
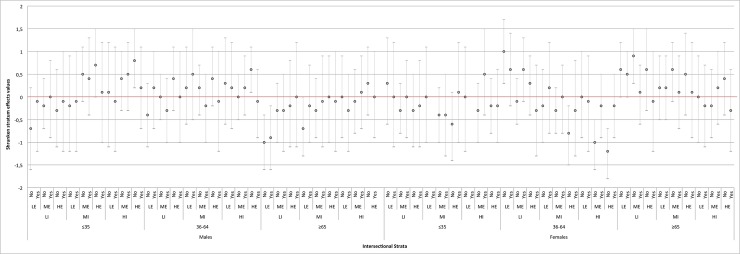
Differences in BMI due to interaction effects (i.e., shrunken stratum effects from the intersectional interaction model, model 2). The intersectional strata are ordered according to the demographic and socioeconomic dimensions used to define the strata. The red horizontal line at value 0 corresponds with the population average BMI. The exact values and the specific definition of the intersectional strata are indicated in the S1 Table. LI: Low Income; MI: Medium Income; HI: High Income. LE: Low Education; ME: Medium Education; HE: High Education.

The predicted stratum effects based on the second intersectional interaction model indicate the strata specific intersectional interaction effects. These effects (Fig 3 and S1 Table) are, as expected, much smaller in absolute value than those based on model 1 (Fig 2 and S1 Table). According to Fig 3 and S1 Table, only nine intersectional strata present conclusive differences, of which five present values that are lower, and four higher, than that predicted by the additive main effects. Furthermore, the stratum with the highest predicted interaction effect is the same as the stratum with the highest predicted overall effect in model 1 (i.e., stratum number 106 consisting of *females between 36 and 64 years*, *who have low income*, *low education and do not live alone)*, but now it showed an average value 1.0 BMI units higher than predicted. Thus, if we base our calculations only on the main effects, model 2 predicts the average BMI in this stratum to be 2.49 (= (25.18−1.16+2.12+0.17+1.71)−26.07) units higher than the overall population average. However, the actual observed average BMI in this stratum is higher because there is a positive interaction of effects between the specific characteristics that define the stratum. On the other hand, in model 2 (S1 Table), stratum number 1 consisting of *females between 36 and 64 years*, *with high income*, *high education and who do not live alone*, presents the largest negative interaction effect (i.e., -1.2 BMI units lower). This negative interaction effect reduces the predicted average BMI of this stratum, which, based only on the sum of the main effects would be -0.07 (= (25.18−1.16+2.12)−26.07) BMI units lower than the population average.

## Discussion

Our study confirms the results from previous traditional analyses of socioeconomic differences in obesity and indicates that, on average, women and individuals living alone have a lower BMI than men and cohabiting people, respectively [11, 72]. It also shows that, on average, BMI increases with age and is higher among individuals with lower levels of income and, especially, lower levels of educational achievement [11, 72]. However, by adopting an intersectional MAIHDA approach [29], our study improves those traditional analyses in several key ways.

### Better mapping of socioeconomic differences

Mapping socioeconomic differences in health is a fundamental task of applied health economics and social epidemiology. However, while traditional studies focus on the identification of socioeconomic gradients based on one or just a few categories of socioeconomic variables (e.g., levels of educational achievement or of income), the intersectional matrix itself provides a much more detailed mapping of the socioeconomic differences in BMI in the population. Our study describes socioeconomic differences between 106 different intersectional strata defined by combinations of categories of age, gender, income, education and habituation (living alone/cohabiting). This approach improves the understanding of the heterogeneity of the socioeconomic distribution of BMI. However, the idea of the existence of such heterogeneity is not new. For instance, Ortiz-Moncada et al [22] observed in 2006 that the prevalence of overweight and obesity in Spain was already higher in men than women. In contrast, the 2017 OECD update report on obesity indicates that by around 2015, in most OECD countries there were more obese women than men [82]. Thus, Spain goes against this general trend. The existence of a socioeconomic gradient in BMI in Spain has also been noted, with regards to educational level and household income [8, 9, 13–16]. Also, the education gradients in BMI are more prominent than the income gradients [8, 13, 14]. From this perspective, the intersectional MAIHDA explicitly provides a more complete picture. In fact, most of the intersectional strata with the lowest BMI levels have medium or high education levels, while some of them are composed of low-income individuals (see for instance the strata located in positions 5, 6, 11, 14 and 16 in the ranking of model 1 in S1 Table).

It has further been shown that, both in Spain [8, 15] and in other countries [27, 83], the educational and income differences in BMI are more pronounced among women than among men. Sarlio-Lähteenkorva et al [28] showed that the socioeconomic gradient in BMI is not manifested among men. In fact, in our study, males with high income and education (see strata 70 and 80 in the ranking of model 1 in S1 Table) present a BMI that is higher than the population mean. Also, men who also have low income but high education (see, in model 1, stratum in 16^th^ position) report a lower BMI than the population average. Our results also confirm the results of Evans et al [33], as we can see that the combination of privileged and marginalized categories can result in effects different to those based on the simple main effects of the separate demographic and socioeconomic variables. This situation can be observed in strata 2, 4, 97 and 104 (^lranking of model 2 in S1 Table).

The construction of the intersectional matrix of strata enables an investigation of BMI levels in groups occupying complex positions of both privilege and disadvantage. This can be seen more clearly among females, since both the most vulnerable and the most protected strata are made up of women, at least in Spain. These females differ with regards to income and educational levels, as the most vulnerable women are those with low income and education.

Ailshire and House [26] also obtained similar results, especially regarding the relevance of educational achievements and income, as less privileged intersectional groups (low-educated and low-income black women) were more affected by increases in weight than more privileged ones (highly educated and high-income white men). However, while the results of Ailshire and House indicate that men are the least affected, our results suggest that women, not men, are those who occupy the position at the protective extreme.

In Raftopoulou’s [24] multilevel study of the Spanish population, the main conclusions are in line with our results, as this analysis again corroborates evidence from the literature on the relationship between socioeconomic variables and BMI, specifically the inverse association between both education and income and BMI.

### The intersectionality approach provides an improved understanding of social stratification in BMI

Intersectional theory has been considered as a highly valid theoretical approach to understanding social stratification [84]. Intersectionality theory conceptualizes social, economic and demographic (or any other) categories as interlocking influences rather than separate dimensions. This type of analysis allows for recognition of the different systems of privilege and disadvantage by means of the assessment of social categories and the possible interaction between the variables that define them [38]. This approach allows us, for instance, to inquire into the societal factor that conditions the high average BMI in females between 36 and 64 years, who have low income, low education and who do not live alone. Our results provide a better understanding of the socioeconomic heterogeneity in BMI and identify the need for further analyses of such intersectional determinants in order to launch appropriate public health interventions. In this way, this study opens the way for a social theoretical reflection while addressing the critique directed at (social) epidemiology concerning the lack of theoretical background in many quantitative empirical analyses and the request for further integration of, and dialogue between, epidemiology and social theory [85]. However, translating intersectional theory into social epidemiology is complicated and challenging [29, 86]. The majority of empirical studies concerned with intersectional theory have been qualitative, and quantitative analyses are still being questioned within intersectionality research [87]. From a classical normative perspective, the intersectionality framework exists prior to and beyond specific research questions, and is not necessarily most appropriately verified or operationalized by means of statistical analyses. In contrast, from a social epidemiological perspective, intersectionality represents a theory that can be empirically investigated through a quantitative analytical approach. Our study, therefore, exists in a space of tension between these stances. This tension will hopefully contribute to ongoing and fruitful dialogue between those traditions, as both share the common goal of eliminating unfair health disparities in society [29].

### The application of MAIHDA within the intersectional framework

A major strength of our study is the application of MAIHDA within the intersectional framework [29, 32, 33], as this methodology provides both technical and conceptual advantages. In the interaction analysis (model 2) we found that, after adjustment for socioeconomic main effects, the interaction effects accounted for 1.9% of BMI variance; so most of the variance located in the intersectional strata level is due to the main effects of the socioeconomic and demographic variables recorded in this study. Indeed, while 14% (= (0.35/2.55)×100) of the variation between strata is attributable to interaction effects (interactive effects), main effects (additive effects) explain 86% of this variability. So, the effect of the intersectional interaction between these social dimensions is smaller than the sum of their main effects. However, the fact the interactive component of the intersectional effect is small does not decrease the value of the intersectional effects, or the merit of intersectional MAIHDA in providing a better mapping of BMI distribution as compared to classical unidimensional analyses. We identified a specific stratum (i.e., stratum number 1 in the ranking of model 2 in the S1 Table) with a relatively large interaction effect, but we note more generally that few strata presented significant interactions effects. Additionally, it should be noted that there are other factors, not included in this analysis, influencing on the BMI and explaining most of its total variability (i.e., 87.65% of the variance in the simple intersectional model 1).

### Avoiding the “tyranny of the averages”

While the “tyranny of the averages” is a rather common phenomenon in epidemiology, it conveys the risk of stigmatization and reducing the effectiveness of public health interventions [55]. As we have explained previously, the ICC is a measure of the discriminatory accuracy of the intersectional strata and indicates to what extent we can know the BMI of an individual by knowing the average BMI of their intersectional strata. In other words, components of variance make it possible to determine if social categorizations provide powerful information about how belonging to a specific intersectional stratum conditions the health outcome of an individual [29]. In our case, the model 1 ICC was 12.4%, which suggests that categorizing the individual by intersectional criteria has some relevance, at least from a public health and social epidemiological perspective. Nevertheless, the moderate clustering of BMI within intersectional strata also indicates a low DA. That is, knowing the average BMI of the intersectional group does not mean we can identify the BMI of the individual with any degree of accuracy. In sum, the moderate intersectional effects (i.e., ICC = 12.4%) suggest that a categorical approach is merited. However, these effects are mainly due to additive rather than to interactive effects.

### Limitations

While most previous studies of socioeconomic position and BMI have taken into account the household rather than individual income, those studies use the educational achievement of the individual instead of the highest educational level existing in the household, as we have done. We believe, however, that household measures in general are better proxies of socioeconomic position, as it is rational to assume that this position is shared by all the individuals in the same household. However, we also performed a sensitivity analysis using the individual educational level and the results were similar.

Another limitation relates to the fact that weight and height are self-reported. Studies show evidence of a strong correlation between self-declared BMI and measured BMI in Spain [88–90]. However, individuals may give incorrect anthropometric information, as underestimation of weight and overestimation of height has been shown to be a generalized behavior in different countries [91–93], especially among women [91] and individuals with a higher BMI [91, 94]. Studies focused on the validity of self-reported anthropometric measures in Spain [88–91] point to differences in misreporting BMI by demographic and socioeconomic characteristics. Women and university graduates in particular have been shown to underestimate their weight, while women, individuals with lower education and those of older age overestimate their height [91]. However, according to Costa-Font et al [15], the difference between self-reported and measured BMI does not affect the results pertaining to inequalities in obesity, such as those related to income, while they do influence prevalence rates. We assume that our results may be underestimated if people, especially in the intersectional strata with the higher BMI average, tended to misestimate weight and height [93]. Unfortunately, as many studies on obesity using data from the Spanish National Health Survey [8, 9, 13, 14, 22], we cannot correct or adjust for the measurement error, but just acknowledge this limitation.

The survey presented a considerable number of missing values, especially with respect to income. As with other studies relating socioeconomic characteristics to obesity/overweight [8, 13–15, 22, 95–98], we simply excluded individuals with one or more missing values rather than pursue, for example, more sophisticated imputation based procedures. This led to 30% of the initial sample being excluded from the final analysis. However, to check for potential selection-bias, we have estimated the probability of income non-response as a function of the remaining variables of our study (gender, age, education, living alone, and BMI) via a logit model (logit estimations are available on request). The pseudo-R-squared was just 0.0066 and the probability of not declaring one’s income decreased with BMI (i.e., Odds Ratio = 0.98). The weaknesses of these predictive relationships lends evidence to the notion that, at least in this sample, those who do not declare their income do not differ systematically from those that do report their income. Thus, we do not expect the exclusion of individuals with missing income from our analysis to alter our results to any great extent. Nevertheless, future analyses might more formally explore this issue via, for example, multiple imputation, but that is beyond the scope of the current work.

Another limitation relates to pregnant women, whose BMI may be higher due to their pregnancy status. While these women are included in the survey, we cannot identify them. Unfortunately, there is no way to solve this problem.

The constructed matrix of analysis was informed by intersectional theory [35, 40]. However, we were limited by the information available in the database, so the intersectional strata were somewhat conditioned by the data availability rather than by theoretical reasons. In addition, our results are influenced by how the strata have been created or defined. In this sense, the creation of new categories within each variable used in this study or even another combination of different variables may alter the results.

The quantitative analysis of intersectional strata requires large databases in order to decrease the number of strata with no or with very few individuals. Therefore, the number of categories in the explanatory variables was limited by the SNHS sample size, since the greater the number of categories, the larger the number of intersectional strata and the lower the number of individuals in each one. In our study, 23 intersectional strata (i.e., 21% of the total strata) contained less than 20 individuals. However, as discussed above, this is not a major limitation since the multilevel regression analysis provides shrunken predictions that take into account the reliability of the sample information.

## Conclusions

Our study confirms the existence of socioeconomic differences in BMI in Spain at the time of the survey. However, these differences show a more complex pattern than that provided by most previous studies focused on one or a few independent socioeconomic dimensions. The socioeconomic context (i.e., the intersectional strata) the individual belongs to conditions their individual BMI to an observable degree (ICC = 12.4%), but only a minor part of this general contextual effect is due to the existence of the interaction of effects (14%) between the variables that define the intersectional strata, the majority being due to additive effects of these variables (86%).

Underlying the intersectional strata heterogeneity, we observed that differences in BMI are particularly notable across levels of educational achievement, especially in women.

While our study gives worthy empirical information, it also presents an innovative and original contribution to research on obesity inequalities in the Spanish population, through the application of a MAIHDA within an intersectional framework [29, 33, 34]. This approach provides a better understanding of the socioeconomic gradient in obesity while at the same time improving the information for policy makers concerned with the prevention of overweight and obesity. According to our results, public policies and actions aiming to tackle obesity should be focused on improving living conditions in order to stop the rapid progress of this chronic disease, since, according to OECD projections, obesity figures will rise continuously up to 2030 [82]. In addition, the OECD report [82] exposes how inequalities in obesity and overweight are widening worldwide. It has also been indicated that in order to avoid such increases, policy design should account for demographic and socioeconomic variables [99]. However, we suggest this policy design should adopt an intersectional perspective that considers the size of the general contextual effects. If it (i.e., the ICC) is large, the intervention should focus on the most disadvantaged intersectional strata. However, if the ICC is small, the focus would be on the entire population as intersectional strata have less significance for BMI. On the basis of our study results, both strategies should be combined. International and national institutions must develop policy programs that integrate a multi-dimensional perspective [71], combining economic, social and prevention actions, directed not only at the individual level, but also at the community level [1, 100]. In addition, broad macro- or meso-level policies can be beneficial across groups, and particularly assist underprivileged groups [101], while avoiding misguided and potentially stigmatizing policies based on targeted intervention [57, 59].

In some European countries, intervention strategies targeted at improving the education of individuals have achieved reductions in obesity rates [102], as investment in education is analogous with investment in health [99]. Hence, governments should implement actions to decrease inequalities pertaining to both income and education, through fiscal, social or employment policies. For instance, public social expenditure, especially that of educational and health services, should be increased, or more progressive taxation should be promoted, enabling the improvement of the pension system, social benefits and working conditions, thus eliminating the barriers to access to a better social welfare. Since obesity is related to low levels of physical activity and a poor quality of diet [1, 6, 100, 103–105], preventive strategies should also promote healthier lifestyles [100, 106] through attempts to tackle sedentary habits and the excessive intake of sugar, salt, junk food and sweetened drinks [82, 100]. Several such interventions are already being implemented in Spain, for example the NAOS Strategy [105, 107, 108], which included many actions with these aims [109], or the tax on sugary drinks introduced in Catalonia in 2017 [110].

In summary, in harmony with the previous study by Evans et al [33], our work provides an improved theoretical and quantitative instrument for documenting BMI disparities compared to traditional studies on (unidimensional) socioeconomic gradients in health. Therefore, we suggest that this approach should become an essential methodological tool in health disparities research [29].

## Supporting information

S1 Table Estimated differences between intersectional strata average BMI values and the overall population average BMI. Predicted strata effects from both models are sorted by values of model 1 from the lowest to the highest values. Values considered conclusive are specified with *. Shaded cells represent the minimum and maximum values of the predicted strata effects for models 1 and 2, respectively.
(PDF)
