# Supplementary material for: Socioeconomic differences in body mass index in Spain: An intersectional multilevel analysis of individual heterogeneity and discriminatory accuracy
Source: PLoS One. 2018 Dec 10;13(12):e0208624. doi: 10.1371/journal.pone.0208624 (PMC6287827; doi:10.1371/journal.pone.0208624)
Supplement: S1 Table — Predicted strata effects from both models are sorted by values of model 1 from the lowest to the highest values. Values considered conclusive are specified with *. Shaded cells represent the minimum and maximum values of the predicted strata effects for models 1 and 2, respectively. (PDF) [file pone.0208624.s001.pdf]

		Gender		Age			Income			Education			Living alone		n (%)	Estimated residues (95% CI) Model 1 Simple intersectional	Estimated residues (95% CI) Model 2 Intersectional interaction
Ranking model 1	Ranking model 2	Males	Females	≤35	36-64	≥65	Low	Medium	High	Low	Medium	High	No	Yes			
1	8		■	■				■				■	■		110 (0.78)	**-3.7 (-4.5 - -2.9)***	-0.6 (-1.4 - 0.1)
2	32		■	■					■			■		■	57 (0.4)	-3.6 (-4.7 - -2.6)*	-0.2 (-1 - 0.6)
3	31		■	■					■			■	■		232 (1.63)	-3.3 (-4 - -2.7)*	-0.2 (-0.8 - 0.4)
4	11		■	■				■			■			■	39 (0.27)	-3 (-4.3 - -1.7)*	-0.4 (-1.3 - 0.5)
5	19		■	■			■					■	■		78 (0.55)	-2.7 (-3.7 - -1.8)*	-0.3 (-1.1 - 0.5)
6	30		■	■			■					■		■	22 (0.16)	-2.7 (-4.2 - -1.2)*	-0.2 (-1.1 - 0.8)
7	20		■	■					■		■		■		215 (1.52)	-2.6 (-3.2 - -1.9)*	-0.3 (-1 - 0.3)
8	1		■		■				■			■	■		546 (3.85)	-2.4 (-2.9 - -1.9)*	**-1.2 (-1.8 - -0.7)***
9	10		■	■				■			■		■		233 (1.64)	-2.4 (-3.1 - -1.8)*	-0.4 (-1 - 0.2)
10	5		■		■			■				■	■		225 (1.59)	-1.9 (-2.5 - -1.2)*	-0.8 (-1.5 - -0.2)*
11	18		■	■			■				■		■		439 (3.09)	-1.8 (-2.3 - -1.3)*	-0.3 (-0.8 - 0.3)
12	23		■		■			■				■		■	12 (0.08)	-1.8 (-3.7 - 0.1)	-0.3 (-1.3 - 0.8)
13	35		■		■				■			■		■	123 (0.87)	-1.8 (-2.6 - -1)*	-0.2 (-0.9 - 0.5)
14	59		■	■			■				■			■	56 (0.39)	-1.8 (-2.9 - -0.7)*	0 (-0.9 - 0.8)
15	72	■		■					■			■		■	37 (0.26)	-1.8 (-3 - -0.5)*	0.2 (-0.7 - 1.1)
16	12	■		■			■					■	■		49 (0.35)	-1.7 (-2.8 - -0.5)*	-0.3 (-1.1 - 0.6)
17	68		■	■				■				■		■	8 (0.06)	-1.7 (-3.8 - 0.5)	0.1 (-1 - 1.2)
18	24		■			■			■			■		■	36 (0.25)	-1.5 (-2.8 - -0.2)*	-0.3 (-1.2 - 0.6)
19	21		■		■		■					■		■	17 (0.12)	-1.4 (-3.1 - 0.3)	-0.3 (-1.3 - 0.7)
20	3		■		■				■		■		■		485 (3.42)	-1.3 (-1.8 - -0.8)*	-1 (-1.6 - -0.5)*
21	6	■		■			■			■			■		40 (0.28)	-1 (-2.2 - 0.2)	-0.7 (-1.6 - 0.2)
22	104	■		■					■			■	■		229 (1.61)	-1 (-1.6 - -0.3)*	0.8 (0.2 - 1.5)*
23	39	■		■			■					■		■	3 (0.02)	-0.9 (-3.6 - 1.7)	-0.1 (-1.2 - 1)
24	40	■		■				■		■				■	4 (0.03)	-0.9 (-3.4 - 1.6)	-0.1 (-1.2 - 1)
25	92	■		■					■		■			■	109 (0.77)	-0.9 (-1.7 - 0)	0.5 (-0.3 - 1.2)
26	94		■	■					■		■			■	27 (0.19)	-0.9 (-2.3 - 0.5)	0.5 (-0.4 - 1.5)
27	26	■		■				■		■			■		9 (0.06)	-0.8 (-2.8 - 1.3)	-0.2 (-1.2 - 0.9)
28	34		■		■				■		■			■	115 (0.81)	-0.8 (-1.6 - -0.1)*	-0.2 (-0.9 - 0.5)
29	50	■		■			■				■			■	51 (0.36)	-0.8 (-1.9 - 0.3)	0 (-0.9 - 0.8)
30	103	■		■				■				■	■		100 (0.7)	-0.7 (-1.6 - 0.2)	0.7 (0 - 1.5)
31	25	■		■			■				■		■		379 (2.67)	-0.6 (-1.1 - 0)	-0.2 (-0.7 - 0.4)
32	41	■		■					■	■				■	4 (0.03)	-0.6 (-3.1 - 1.9)	-0.1 (-1.2 - 1.1)
33	42	■			■			■				■		■	7 (0.05)	-0.6 (-2.8 - 1.6)	-0.1 (-1.2 - 1)
34	43	■			■				■			■		■	98 (0.69)	-0.6 (-1.4 - 0.3)	-0.1 (-0.9 - 0.6)
35	61		■	■					■	■			■		2 (0.01)	-0.6 (-3.4 - 2.1)	0 (-1.2 - 1.1)
36	87	■		■					■		■		■		202 (1.42)	-0.6 (-1.2 - 0.1)	0.4 (-0.3 - 1)
37	38	■		■			■			■				■	4 (0.03)	-0.5 (-3 - 2)	-0.1 (-1.2 - 1)
38	45	■				■		■				■		■	5 (0.04)	-0.5 (-2.9 - 1.9)	-0.1 (-1.2 - 1)
39	49		■			■	■					■		■	6 (0.04)	-0.5 (-2.8 - 1.9)	-0.1 (-1.2 - 1)

		Gender		Age			Income			Education			Living alone		n (%)	Estimated residues (95% CI) Model 1 Simple intersectional	Estimated residues (95% CI) Model 2 Intersectional interaction
Ranking model 1	Ranking model 2	Males	Females	≤35	36-64	≥65	Low	Medium	High	Low	Medium	High	No	Yes			
40	60		■	■				■		■			■		7 (0.05)	-0.5 (-2.7 - 1.8)	0 (-1 - 1.1)
41	62		■		■			■			■			■	106 (0.75)	-0.5 (-1.3 - 0.4)	0 (-0.8 - 0.7)
42	86	■		■				■			■			■	51 (0.36)	-0.4 (-1.6 - 0.7)	0.4 (-0.4 - 1.3)
43	22		■		■			■			■		■		600 (4.23)	-0.3 (-0.7 - 0.2)	-0.3 (-0.8 - 0.2)
44	48		■		■				■	■				■	12 (0.08)	-0.3 (-2.2 - 1.5)	-0.1 (-1.2 - 0.9)
45	91	■		■				■			■		■		247 (1.74)	-0.3 (-0.9 - 0.3)	0.5 (-0.1 - 1.1)
46	58		■	■			■			■				■	2 (0.01)	-0.2 (-3 - 2.5)	0 (-1.1 - 1.2)
47	65	■		■				■				■		■	3 (0.02)	-0.1 (-2.7 - 2.5)	0.1 (-1 - 1.2)
48	71		■			■		■				■		■	10 (0.07)	-0.1 (-2.1 - 2)	0.1 (-0.9 - 1.2)
49	85		■		■		■					■	■		133 (0.94)	0 (-0.8 - 0.7)	0.3 (-0.4 - 0.9)
50	90		■			■			■			■	■		83 (0.58)	0 (-0.9 - 1)	0.4 (-0.4 - 1.2)
51	36		■			■			■	■				■	37 (0.26)	0.1 (-1.3 - 1.4)	-0.2 (-1.1 - 0.7)
52	37		■			■			■		■		■		105 (0.74)	0.1 (-0.7 - 0.9)	-0.2 (-0.9 - 0.6)
53	52	■			■		■					■		■	15 (0.11)	0.1 (-1.6 - 1.9)	0 (-1 - 1)
54	57	■				■			■			■		■	30 (0.21)	0.1 (-1.3 - 1.5)	0 (-0.9 - 1)
55	66	■		■					■	■			■		4 (0.03)	0.1 (-2.4 - 2.7)	0.1 (-1.1 - 1.2)
56	70		■			■		■			■			■	89 (0.63)	0.2 (-0.7 - 1.1)	0.1 (-0.7 - 0.9)
57	81		■			■			■		■			■	61 (0.43)	0.2 (-0.8 - 1.2)	0.2 (-0.6 - 1)
58	84		■	■			■			■			■		26 (0.18)	0.2 (-1.2 - 1.7)	0.3 (-0.6 - 1.3)
59	27	■			■			■			■			■	97 (0.68)	0.4 (-0.5 - 1.2)	-0.2 (-1 - 0.5)
60	33		■		■			■		■			■		59 (0.42)	0.4 (-0.7 - 1.5)	-0.2 (-1 - 0.6)
61	47		■		■		■				■		■		845 (5.95)	0.4 (0 - 0.9)	-0.1 (-0.6 - 0.4)
62	54	■				■	■					■		■	7 (0.05)	0.5 (-1.7 - 2.8)	0 (-1.1 - 1.2)
63	55	■				■		■				■	■		32 (0.23)	0.5 (-0.8 - 1.9)	0 (-1 - 0.9)
64	28	■				■	■					■	■		25 (0.18)	0.6 (-0.9 - 2.1)	-0.2 (-1.1 - 0.8)
65	63		■		■				■	■			■		27 (0.19)	0.6 (-0.9 - 2)	0 (-0.9 - 1)
66	69		■			■	■				■			■	170 (1.2)	0.6 (-0.1 - 1.3)	0.1 (-0.6 - 0.7)
67	96		■			■		■				■	■		45 (0.32)	0.6 (-0.6 - 1.8)	0.5 (-0.4 - 1.4)
68	13	■			■		■				■			■	130 (0.92)	0.7 (0 - 1.5)	-0.3 (-1 - 0.4)
69	44	■				■		■			■			■	21 (0.15)	0.7 (-0.9 - 2.3)	-0.1 (-1.1 - 0.9)
70	97	■			■				■			■	■		598 (4.21)	0.7 (0.3 - 1.2)*	0.6 (0.1 - 1.1)*
71	15	■				■	■				■			■	39 (0.27)	0.8 (-0.4 - 2)	-0.3 (-1.2 - 0.5)
72	64		■			■			■	■			■		27 (0.19)	0.8 (-0.6 - 2.3)	0 (-1 - 0.9)
73	77	■			■				■		■			■	242 (1.71)	0.8 (0.2 - 1.4)*	0.2 (-0.4 - 0.9)
74	89	■			■			■				■	■		176 (1.24)	0.8 (0.1 - 1.5)*	0.4 (-0.2 - 1.1)
75	99		■		■		■				■			■	126 (0.89)	0.8 (0 - 1.6)	0.6 (-0.1 - 1.3)
76	78		■		■			■		■				■	15 (0.11)	0.9 (-0.9 - 2.7)	0.2 (-0.8 - 1.2)
77	17	■				■			■	■				■	50 (0.35)	1 (-0.2 - 2.1)	-0.3 (-1.2 - 0.5)
78	53	■			■				■		■		■		527 (3.71)	1 (0.5 - 1.5)*	0 (-0.5 - 0.5)

		Gender		Age			Income			Education			Living alone		n (%)	Estimated residues (95% CI)<br><br>Model 1 Simple intersectional	Estimated residues (95% CI)<br><br>Model 2 Intersectional interaction
**Ranking model 1**	**Ranking model 2**	**Males**	**Females**	**≤35**	**36-64**	**≥65**	**Low**	**Medium**	**High**	**Low**	**Medium**	**High**	**No**	**Yes**			
79	80		■			■		■		■				■	110 (0.78)	1 (0.2 - 1.8)*	0.2 (-0.5 - 0.9)
80	83	■				■			■			■	■		86 (0.61)	1 (0.1 - 1.9)*	0.3 (-0.4 - 1.1)
81	67	■				■			■		■			■	54 (0.38)	1.1 (0 - 2.2)	0.1 (-0.7 - 0.9)
82	4	■				■	■			■				■	152 (1.07)	1.2 (0.5 - 2)*	-0.9 (-1.6 - -0.2)*
83	102		■			■		■			■		■		203 (1.43)	1.2 (0.6 - 1.9)*	0.6 (-0.1 - 1.2)
84	16	■				■		■			■		■		182 (1.28)	1.3 (0.6 - 2)*	-0.3 (-0.9 - 0.4)
85	46	■				■			■		■		■		130 (0.92)	1.3 (0.5 - 2)*	-0.1 (-0.8 - 0.6)
86	88	■			■		■					■	■		122 (0.86)	1.3 (0.5 - 2.1)*	0.4 (-0.3 - 1.1)
87	101		■			■	■					■	■		36 (0.25)	1.3 (0 - 2.6)	0.6 (-0.3 - 1.5)
88	29	■				■		■		■				■	50 (0.35)	1.4 (0.3 - 2.5)*	-0.2 (-1 - 0.7)
89	75	■			■			■			■		■		645 (4.55)	1.4 (0.9 - 1.8)*	0.2 (-0.4 - 0.7)
90	7	■				■		■		■			■		162 (1.14)	1.5 (0.8 - 2.2)*	-0.7 (-1.3 - 0)
91	76	■			■				■	■				■	28 (0.2)	1.5 (0.1 - 2.9)*	0.2 (-0.7 - 1.2)
92	79		■			■		■		■			■		137 (0.97)	1.5 (0.8 - 2.3)*	0.2 (-0.5 - 0.9)
93	98		■		■		■			■				■	73 (0.51)	1.7 (0.8 - 2.7)*	0.6 (-0.2 - 1.4)
94	2	■				■	■			■			■		264 (1.86)	1.8 (1.2 - 2.4)*	-1 (-1.6 - -0.4)*
95	9	■			■		■			■			■		114 (0.8)	1.8 (0.9 - 2.6)*	-0.4 (-1.1 - 0.3)
96	14	■				■	■				■		■		218 (1.54)	1.8 (1.1 - 2.4)*	-0.3 (-1 - 0.3)
97	51	■			■		■				■		■		769 (5.42)	1.8 (1.3 - 2.2)*	0 (-0.5 - 0.5)
98	95		■			■	■			■				■	512 (3.61)	1.8 (1.3 - 2.3)*	0.5 (0 - 1)
99	56	■				■			■	■			■		42 (0.3)	2 (0.8 - 3.2)*	0 (-0.9 - 0.9)
100	73	■			■		■			■				■	67 (0.47)	2 (1 - 3)*	0.2 (-0.7 - 1)
101	105		■			■	■				■		■		261 (1.84)	2.1 (1.5 - 2.7)*	0.9 (0.3 - 1.5)*
102	74	■			■			■		■			■		67 (0.47)	2.2 (1.2 - 3.2)*	0.2 (-0.6 - 1.1)
103	82	■			■				■	■			■		25 (0.18)	2.2 (0.7 - 3.7)*	0.3 (-0.6 - 1.3)
104	93	■			■			■		■				■	20 (0.14)	2.5 (0.9 - 4.1)*	0.5 (-0.5 - 1.5)
105	100		■			■	■			■			■		249 (1.75)	2.5 (1.9 - 3.1)*	0.6 (0 - 1.2)
106	106		■		■		■			■			■		120 (0.85)	**2.6 (1.8 - 3.4)***	**1 (0.3 - 1.7)***
107	107		■	■				■		■				■	-	-	-
108	108		■	■					■	■				■	-	-	-
